# Paromomycin targets HDAC1-mediated SUMOylation and IGF1R translocation in glioblastoma

**DOI:** 10.3389/fphar.2024.1490878

**Published:** 2024-12-11

**Authors:** Zhong Min, Yuejie Guo, Luo Ning

**Affiliations:** The First People’s Hospital of Chenzhou, Chenzhou, Hunan, China

**Keywords:** glioblastoma multiforme, Paromomycin, HDAC1, SUMOylation, IGF1R, drug screening

## Abstract

**Objective:**

This study investigates the effects of Paromomycin on SUMOylation-related pathways in glioblastoma (GBM), specifically targeting HDAC1 inhibition.

**Methods:**

Using TCGA and GTEx datasets, we identified SUMOylation-related genes associated with GBM prognosis. Molecular docking analysis suggested Paromomycin as a potential HDAC1 inhibitor. *In vitro* assays on U-251MG GBM cells were performed to assess Paromomycin’s effects on cell viability, SUMOylation gene expression, and IGF1R translocation using CCK8 assays, qRT-PCR, and immunofluorescence.

**Results:**

Paromomycin treatment led to a dose-dependent reduction in GBM cell viability, colony formation, and migration. It modulated SUMO1 expression and decreased IGF1R nuclear translocation, an effect reversible by the HDAC1 inhibitor Trochostatin A (TSA), suggesting Paromomycin’s involvement in SUMO1-regulated pathways.

**Conclusion:**

This study highlights Paromomycin’s potential as a therapeutic agent for GBM by targeting HDAC1-mediated SUMOylation pathways and influencing IGF1R translocation, warranting further investigation for its clinical application.

## Introduction

Glioblastoma Multiforme (GBM) is the most aggressive and malignant type of brain tumor in adults, predominantly originating in the central nervous system (Banu, 2019; Grochans et al., 2022). This rapidly progressing tumor is characterized by its high malignancy and resistance to conventional treatments, leading to substantial physical and psychological burdens for patients and their families (Yalamarty et al., 2023; Tian et al., 2024). Accounting for nearly 50% of all primary brain tumors, GBM is the most prevalent malignant brain tumor in adults, with the majority of diagnoses occurring in middle-aged and elderly individuals (Davis, 2016; Stylli, 2021). Accounting for nearly 50% of all primary brain tumors, GBM is the most prevalent malignant brain tumor in adults, with the majority of diagnoses occurring in middle-aged and elderly individuals (Grech et al., 2020; Ostrom et al., 2018). Additionally, the incidence is slightly higher among males and certain demographic groups, such as African Americans (Tosakoon et al., 2023). GBM is associated with high mortality rates and requires extensive healthcare resources, including surgery, radiation, chemotherapy, and long-term rehabilitation (Dasari et al., 2020; Aly et al., 2019). GBM is associated with high mortality rates and requires extensive healthcare resources, including surgery, radiation, chemotherapy, and long-term rehabilitation (Elsaid et al., 2020). This challenging clinical landscape underscores the critical need for advancing our understanding of GBM’s underlying biology and developing novel therapeutic strategies.

The complexity and heterogeneity of GBM stem from various genetic mutations, chromosomal aberrations, and downregulation of tumor suppressor genes (Nakada et al., 2011; Kesari, 2011). These genetic factors are pivotal in the initiation, progression, and treatment resistance of GBM (Nguyen et al., 2021; Chen et al., 2022). This challenging clinical landscape underscores the critical need for advancing our understanding of GBM’s underlying biology and developing novel therapeutic strategies (Nguyen et al., 2021; Chen et al., 2022). Additionally, disruptions to the blood-brain barrier (BBB) facilitate cellular infiltration that enhances tumor invasiveness while impeding therapeutic agent delivery. Despite the conventional approach of tumor resection followed by adjuvant radiation and chemotherapy, most GBM tumors tend to recur, often in multiple regions, complicating effective treatment. Challenges in achieving complete surgical resection and the development of resistance to radiotherapy and chemotherapy further impede patient outcomes. The median survival for GBM patients remains approximately 12–15 months post-diagnosis, although survival can vary depending on individual patient characteristics. Consequently, there is an urgent need to identify and target novel therapeutic pathways to improve GBM treatment outcomes. The manipulation of biomolecules, in combination with environmental exposure, can provoke diverse biological responses, and such interactions—documented across various experimental contexts—highlight promising therapeutic targets (Du and Liu, 2024; McNerney and Styczynski, 2018; Cheng et al., 2013). The synergy of pharmacotherapy and bioinformatics has further amplified the potential of modern medical research, with large-scale bioinformatics databases elucidating associations between physiological markers and long-term health outcomes, offering valuable data to inform clinical decision-making (Liao et al., 2023; Tsuji et al., 2023; Wu W-T. et al., 2021; Asano, 2018; Srivastava and Kumar, 2024; Li, 2015; Behl et al., 2021). Additionally, animal models that replicate disease physiology are essential for validating therapeutic efficacy and generating robust supporting data (Pechanova, 2020; McGonigle and Ruggeri, 2014). In clinical practice, shared decision-making tools and checklists have proven effective in increasing patient engagement and satisfaction, particularly in drug selection and treatment planning (Li et al., 2023; Wieringa et al., 2019; Slyer, 2022). Furthermore, the rapid development of artificial intelligence (AI) is advancing healthcare by enhancing decision-making accuracy through sophisticated algorithms and data-driven analysis, paving the way for more personalized treatment recommendations (Shan et al., 2024; Hao et al., 2024).

Gene-environment interaction studies, a subset of bioinformatics methodologies, have proven effective in analyzing survival data derived from large-scale genomic analyses. These approaches have revealed molecular pathways potentially responsible for a variety of complex conditions (Wang et al., 2022). Recent advancements in bioinformatics and molecular biology have significantly enriched our understanding of GBM, revealing its intricate molecular basis through genomic, proteomic, and metabolomic investigations. This progress has facilitated the emergence of innovative therapeutic strategies (Kumar et al., 2008; Kaynar et al., 2021). Ongoing research focuses on personalized medicine, targeted therapies, and immunotherapies aimed at overcoming resistance to radiation and enhancing therapeutic efficacy while improving the overall quality of life for patients (Yang and Cai, 2023; Wayteck et al., 2014). A comprehensive understanding of GBM’s epidemiology, biological characteristics, and treatment challenges is essential for optimizing curative strategies (Aldoghachi et al., 2022; Kim and Kim, 2020). Future investigations should prioritize the exploration of molecular pathways that drive tumorigenesis and treatment resistance, with the goal of translating these findings into improved survival rates and quality of life for patients (Ou et al., 2020; Wu W. et al., 2021).

Among promising therapeutic targets, post-translational modifications (PTMs) such as SUMOylation (Small Ubiquitin-like Modifier modification) have garnered significant attention (Woo and Abe, 2010; Zhao et al., 2021). SUMOylation regulates numerous cellular functions, including the cell cycle, DNA damage response, and apoptosis (Han et al., 2018). Studies indicate that elevated SUMOylation activity may promote GBM development and progression by modulating these pathways, positioning SUMOylation as a viable target for therapeutic intervention (Fox et al., 2019). At the genomic level, extensive evidence supports the link between genetic variations and the development and progression of GBM (Backes et al., 2015; Pasche and Myers, 2009). Recent studies have identified specific biomarkers and treatment strategies, which are generating new opportunities for GBM management (Lynes et al., 2020). For instance, mutations in the IDH1/IDH2 genes are prevalent in GBM and have been associated with metabolic changes that impact tumor cell proliferation and survival (Miller et al., 2017; Yan et al., 2009). Furthermore, alterations or deletions in the TP53 gene lead to the loss of p53 protein function, disrupting cell cycle control and hindering DNA repair mechanisms (Monti et al., 2020; Vaddavalli and Schumacher, 2022). These genetic alterations serve as cellular markers for GBM diagnosis and classification and may represent potential therapeutic targets (Szopa et al., 2017). Recent advancements in bioinformatics have significantly transformed disease research, enabling comprehensive multi-omics analyses that provide critical insights into the molecular mechanisms underlying disease progression (Glass, 2023; Sun and Hu, 2016). Recent advancements in bioinformatics have significantly transformed disease research, enabling comprehensive multi-omics analyses that provide critical insights into the molecular mechanisms underlying disease progression (Yan et al., 2022; Chen et al., 2020; Cavill et al., 2016). This integrated analysis has become indispensable in disease diagnosis, prognosis assessment, and treatment evaluation, thus reinforcing the foundations of precision medicine (Guo Z. et al., 2023; Huang L. et al., 2022; Huang et al., 2013; Wang et al., 2005; Huang et al., 2015; Yang et al., 2024). This study aims to assess the effects of Paromomycin on SUMOylation-related pathways in GBM. By combining bioinformatics analysis, molecular docking, and *in vitro* validation, this research seeks to contribute to the development of novel targeted therapies for GBM.

## Materials and methods

### Expression profiling of SUMOylation-related genes in pan-cancer analysis

In this research, we conducted a detailed evaluation of SUMOylation-related gene expression across multiple cancer types. To assess variations in gene expression between different cancers and adjacent non-tumor tissues, we applied the Wilcoxon Rank Sum Test. To further analyze expression differences within each cancer type between malignant and adjacent non-tumor tissues, we utilized the Wilcoxon Signed Rank Test, a non-parametric method for dependent samples. Consistency was maintained by merging TPM expression data from GTEx normal samples with corresponding TCGA tumor data, using the tcgasandbox_RSEM_gene_tpm and gtexsandbox_RSEM_gene_tpm datasets from the UCSC Xena database. We standardized the data by converting it to Z-scores, ensuring uniform comparisons across tumor subtypes. For comparative analysis of expression levels between tumor and non-tumor tissues in TCGA and GTEx datasets, we focused on the GBM dataset, employing the Wilcoxon Rank Sum Test. This statistical method, also known as the Mann-Whitney U Test, is a reliable tool for assessing differences between two independent samples, testing the hypothesis regarding the median comparability of two populations at a significance level of α = 0.05.

### Promoter methylation analysis of SUMOylation-related genes

This analysis focused on examining methylation levels in specific genomic regions, including TSS1500 (spanning 200 to 1,500 bp upstream of the TSS), TSS200 (within 200 bp of the TSS), the first exon, and the 5′untranslated region (5′UTR). Median methylation levels across these regions were calculated for each sample to assess cumulative methylation. A Spearman correlation analysis was also conducted to determine potential associations between methylation levels and gene expression. The non-parametric Spearman rank correlation coefficient was employed to analyze this association without assuming data normality, treating methylation as the independent variable and gene expression as the dependent variable. The Wilcoxon Rank Sum Test was additionally used to compare methylation patterns between tumor and non-tumor groups, allowing for distributional comparisons across independent groups without presuming normality.

### ATAC-seq analysis of SUMOylation-related genes

Using the ChIPseeker package in R, we examined ATAC-seq data of SUMOylation-related genes. Peaks were annotated at gene promoters around the transcription start site (TSS) with parameters set to tssRegion = c (−3,000, 3,000) to capture areas extending 3,000 bp upstream of the TSS and covering up to +3 kb downstream. This approach is a standard method to assess transcription factor binding, histone modifications, and other genomic interactions around the TSS. Chromosomal distributions of ATAC-seq peaks were visualized using the covplot function, presenting peak locations across chromosomes, along with relevant genomic distances and tumor types.

### Genomic characterization of SUMOylation-related genes in pan-cancer studies

We retrieved copy number variation (CNV) and DNA methylation data from TCGA across various cancers. Data matrices were organized with rows representing samples and columns as individual genes or genomic loci, undergoing quality control to remove low-quality samples and normalize for technical variation. In OSCC samples, CNV analysis was performed using GISTIC and CNAnorm, categorizing genes into amplified or deleted based on CNV levels. DNA methylation in promoter regions of SUMOylation-related genes was assessed in both tumor and normal tissues through the UALCAN platform. Methylation patterns across different cancers were further analyzed using the MethSurv database, aiming to establish any association between methylation and cancer incidence. Mutation Annotation Files (MAF) were downloaded from TCGA using the “TCGAbiolinks” R package. Tumor mutation burden (TMB), indicating genomic instability potentially related to immunotherapy response, was calculated with the “maftool” package. We examined the relationship between SUMOylation gene expression and CNV, DNA methylation, and TMB, using statistical analyses, correlation studies, survival plots, and other computational tools in R to explore the impact of these genetic features on tumor progression and patient outcomes.

### GSEA enrichment analysis across pan-cancer types

Expression data for several cancer types, including both tumor and adjacent normal samples, were collected from The Cancer Genome Atlas (TCGA) database, incorporating RNA-seq and microarray sources. Following normalization, samples and probes that did not meet predefined quality criteria were removed from further analysis. Differential expression analysis was performed using the R package “limma,” which provides methods for data normalization, background correction, and statistical testing to identify significantly altered genes. Key genes were determined based on log2 fold change (log2FC) to represent expression differences, alongside the P-value to assess statistical significance. Gene Set Enrichment Analysis (GSEA) was carried out with the R package “clusterProfiler,” allowing us to interpret biological functions of differentially expressed genes through pathway databases like KEGG, Gene Ontology (GO), and Reactome. The Enrichment Score (ES), ranging from 0 to 1, was calculated to quantify the association between gene expression and specific biological processes, facilitating pathway relevance assessment. Visualizations, including bar graphs, scatter plots, and heatmaps, were generated using the “ggplot2” package in R, known for its flexibility in data visualization.

### Tumor Prognosis analysis

The TCGA database provided RNA-seq and microarray data, alongside clinical and survival information across various cancer types. Using the “limma” package, we analyzed differential gene expression to identify genes with significant up- or downregulation in tumor samples compared to paired normal tissues. Our focus was on SUMOylation-related genes with expression levels potentially linked to overall survival (OS). To investigate these genes’ impact on OS, we used a Cox proportional hazards model in R’s “survival” package. Kaplan-Meier survival curves were generated and compared using the log-rank test to illustrate survival differences between low- and high-expression groups, with plots created using the “survminer” package, which allows for clear visualization of survival outcomes.

### Developing a prognostic model for SUMOylation-related genes in GBM

To evaluate the diagnostic performance of the ssGSEAscore in differentiating tumor samples from normal samples, ROC analysis was conducted using the “pROC” package, which calculated the area under the curve (AUC) and plotted a smooth ROC curve along with a 95% confidence interval. The ssGSEAscore was determined using the “gsva” package’s gsva function with the “ssgsea” method. Expression data for this study were acquired from the TCGA dataset, specifically the EBPlusPlusAdjustPANCAN_IlluminaHiSeq_RNASeqV2 dataset, accessible through PanCanAtlas in the geneExp.tsv file. This file was generated via the Firehose pipeline using MapSplice and RSEM, with standardization by setting the upper quartile value to 1,000. The Wilcoxon Rank Sum Test was used to compare ssGSEAscore expression between tumor and normal tissues in the GBM dataset, while the Wilcoxon Signed Rank Test assessed ssGSEAscore in tumor tissues relative to adjacent normal tissues. Calibration curves were also constructed to show alignment between predicted and actual outcomes in tumor classification, and goodness-of-fit tests were applied to assess model accuracy. Furthermore, ssGSEAscore variations in different GBM stages were examined using the Wilcoxon Rank Sum Test, and the Kruskal-Wallis Rank Sum Test compared ssGSEAscore expression among GBM progression phases.

### Survival prognosis analysis of SUMOylation-related genes in GBM using ssGSEA

To assess overall survival (OS), disease-specific survival (DSS), and progression-free interval (PFI) based on SUMOylation-related genes, we conducted Kaplan-Meier survival analysis using the “survival” package in R. The ssGSEAscore thresholds were determined with the “survminer” package, ensuring that the ratio between groups did not drop below 0.3. Each survival analysis was executed using the survfit function, with high- and low-score groups compared via a log-rank test. For Cox survival analysis, we conducted a meta-analysis with inverse variance weighting, including data from sixteen qualifying studies, and measured hazard ratios (HR) in logarithmic form. HRs were divided into two categories: less than 1, indicating a tumor-suppressive effect, and greater than 1, suggesting an oncogenic role. While this stratification helped differentiate tumor impacts, it did not capture the full range of regulatory functions associated with the targeted genes. Statistical analyses and visual representations were generated using the Meta package in R (version 4.3.2). Additionally, each gene underwent univariate Cox survival analysis through the “survival” package, employing the Cox proportional hazards model via the coxph () function. Forest plots were created using the “forestplot” package to illustrate HRs and their 95% confidence intervals (CIs).

### Core protein drug sensitivity screening

Virtual screening is an efficient technique in drug discovery, enabling prediction of a compound’s biological activity by modeling interactions with biological targets, thus reducing both time and costs associated with drug research. In our study, we obtained 3D structures of 321 FDA-approved drugs from the ZINC database. Core protein domains were downloaded in PDB format from the Protein Data Bank (PDB). Screening was performed with the Libdock tool in Discovery Studio 2019 (DS 2019). Prior to screening, PDB structures underwent preprocessing: water molecules were removed, receptor protein structures were optimized, and energy minimization was applied to both proteins and ligands. Key amino acid residues were set to appropriate ionization states, tautomers were generated, and non-polar hydrogen atoms were removed. Important atomic charges were assigned using the Gasteiger-Marsili approach. Molecular docking was employed to explore potential interactions between candidate drugs and the binding sites of target proteins, assessing compatibility with the protein binding regions. These findings provide a foundation for drug design refinement and subsequent experimental validation.

### CCK8 proliferation activity assay

For cell proliferation assays, U-251MG cells were seeded at a density of 5 × 10³ cells per well in 96-well plates. Different concentrations of Paromomycin were introduced to each well, and cells were incubated for 48 h. Following this, 10 μL of CCK-8 solution was added to each well, with an additional 2-h incubation period. Absorbance was measured at 450 nm using a microplate reader. For TSA treatment, cells were incubated for 24 h in fresh culture medium containing 100 nM of TSA. Control wells received fresh medium with 0.1% DMSO under the same conditions. The experiment was repeated three times to ensure reproducibility.

### qRT-PCR

To extract total RNA, 1 mL of Trizol reagent was added per well, and the solution was transferred to 1.5-mL tubes for 10 min of lysis. After sonication, 200 μL of chloroform was added, followed by centrifugation at 12,000 rpm for 15 min at 4°C. The supernatant was collected, mixed with 400 μL of isopropanol, and centrifuged to isolate the RNA pellet, which was then dissolved in 20 μL of DEPC water. RNA was reverse-transcribed into cDNA under specific temperature conditions for qRT-PCR.

### Immunofluorescence

Cell slides were prepared and incubated with a bovine serum albumin (BSA) solution for 1 h to block non-specific binding. After blocking, the slides were rinsed and incubated overnight at 4°C with either a 1:250 dilution of anti-CAS3 or a 1:100 dilution of anti-SUMO1 antibody. The following day, slides were washed in PBS and then incubated with a fluorescein-labeled secondary antibody for 2 h at room temperature. Cell nuclei were stained with DAPI, and after a final rinse, the slides were fixed for imaging. Fluorescence microscopy was used to visualize the expression levels of CAS3 and SUMO1 proteins across different cell clusters.

### Colony formation assay for U-251MG cells

The colony-forming ability of U-251MG glioblastoma cells after Paromomycin treatment was evaluated. Cells were maintained in Dulbecco’s Modified Eagle Medium (DMEM) (Gibco BRL, MD, United States) with 10% fetal bovine serum (FBS) (HyClone). U-251MG cells were seeded at 500 cells per well in six-well plates and allowed to adhere overnight. The following day, cells were treated with the appropriate concentration of Paromomycin or vehicle control (DMSO), with fresh treatment medium replaced every 3–4 days. After 10–14 days, during which colonies became visible, the medium was removed, and cells were gently rinsed twice with PBS. The cells were then fixed with 4% paraformaldehyde for 15 min at room temperature. After fixation, colonies were stained with 0.5% crystal violet solution for 20 min, rinsed with distilled water to remove excess stain, and air-dried.

### Statistical analyses

The statistical analyses were performed using GraphPad Prism version 8.0 (GraphPad Software, La Jolla, CA, United States). In order to guarantee the dependability of the outcomes, all trials were conducted three times. The data are reported as the average value plus or minus the standard deviation (SD). A two-tailed Student’s t-test was employed to compare two samples. The data distribution was analyzed for normality using the Shapiro-Wilk test, and the equality of variances was verified using Levene’s test. In order to compare more than two groups, we used one-way analysis of variance (ANOVA) followed by Tukey’s *post hoc* test to discover particular differences between the groups. A p-value less than or equal to 0.05 was deemed to be statistically significant.

## Results

### Relationship between the expression of SUMOylation-related genes and tumor prognosis

**FIGURE 1:**
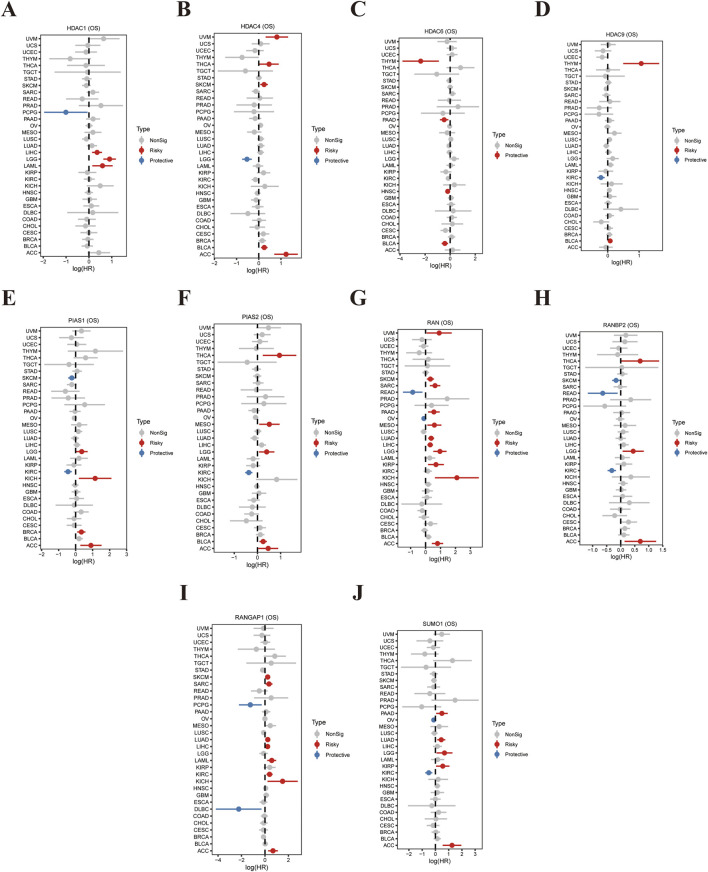
Correlation Between SUMOylation-Related Gene Expression and Tumor Prognosis. **(A)** The forest plot presents hazard ratios (HR) and 95% confidence intervals (CI) for HDAC1 expression and its association with overall survival (OS) across various cancer types. Each line represents a specific cancer, with red indicating a negative (risk) factor and blue indicating a positive (protective) factor. **(B)** Similar analysis for HDAC4, displaying its relevance to OS across multiple cancers, where HR and CI indicate the impact of HDAC4 expression on patient outcomes. **(C)** Depicts the influence of HDAC6 expression on OS, with corresponding HR and CI values highlighting its role in cancer prognosis. **(D)** A validation analysis utilizing an independent dataset to confirm or compare the effects of HDAC6 expression on OS. **(E)** Shows the relationship between PIAS1 expression and OS across various malignancies, demonstrating its potential role in cancer progression. **(F)** Displays the impact of PIAS2 expression on OS, with HR and CI values reflecting its prognostic significance in different cancers. **(G)** This forest plot illustrates the association between RAN expression and OS across all analyses. **(H)** Shows the influence of RANBP2 expression on OS, with HR and CI values indicating its predictive power for tumor outcomes. **(I)** Analyzes the relationship between RANGAP1 expression and OS, highlighting its potential role in enhancing survival benefits in cancer patients. **(J)** Presents HR and CI values for SUMO1 expression, showcasing its correlation with patient prognosis and impact on OS across various cancer types.

This study investigates the correlation between the expression levels of ten genes associated with SUMOylation (HDAC1, HDAC4, HDAC9, PIAS1, PIAS2, RAN, RANBP2, SUMO1, RANGAP1, SUMO1) and overall survival (OS) across various cancer types, as illustrated in Figure 1. The forest plots display hazard ratios (HR) and 95% confidence intervals (CI) for each gene in different malignancies. Figure 1A shows a strong association between elevated HDAC1 expression and increased risk, alongside unfavorable outcomes in several cancer types. Similarly, Figure 1B highlights that high HDAC4 expression is a significant prognostic indicator. Consistent findings across multiple datasets, including Figures 1C, D, reveal a robust association between high HDAC6 expression and poor OS. PIAS1 (Figure 1E) and PIAS2 (Figure 1F) show mixed outcomes, suggesting that their prognostic roles vary by cancer type. Increased expression of RAN (Figure 1G) is linked to reduced OS, while RANBP2 (Figure 1H) and RANGAP1 (Figure 1I) demonstrate protective effects, with higher expression associated with improved survival. SUMO1 (Figure 1J) appears to function as both a risk factor and a protective factor, contingent on the specific cancer type. These findings underscore the prognostic significance of SUMOylation-related genes, highlighting their potential as therapeutic targets and prognostic indicators in cancer.

### Relationship between the expression of SUMOylation-Related genes and Tumor Prognosis

**FIGURE 2:**
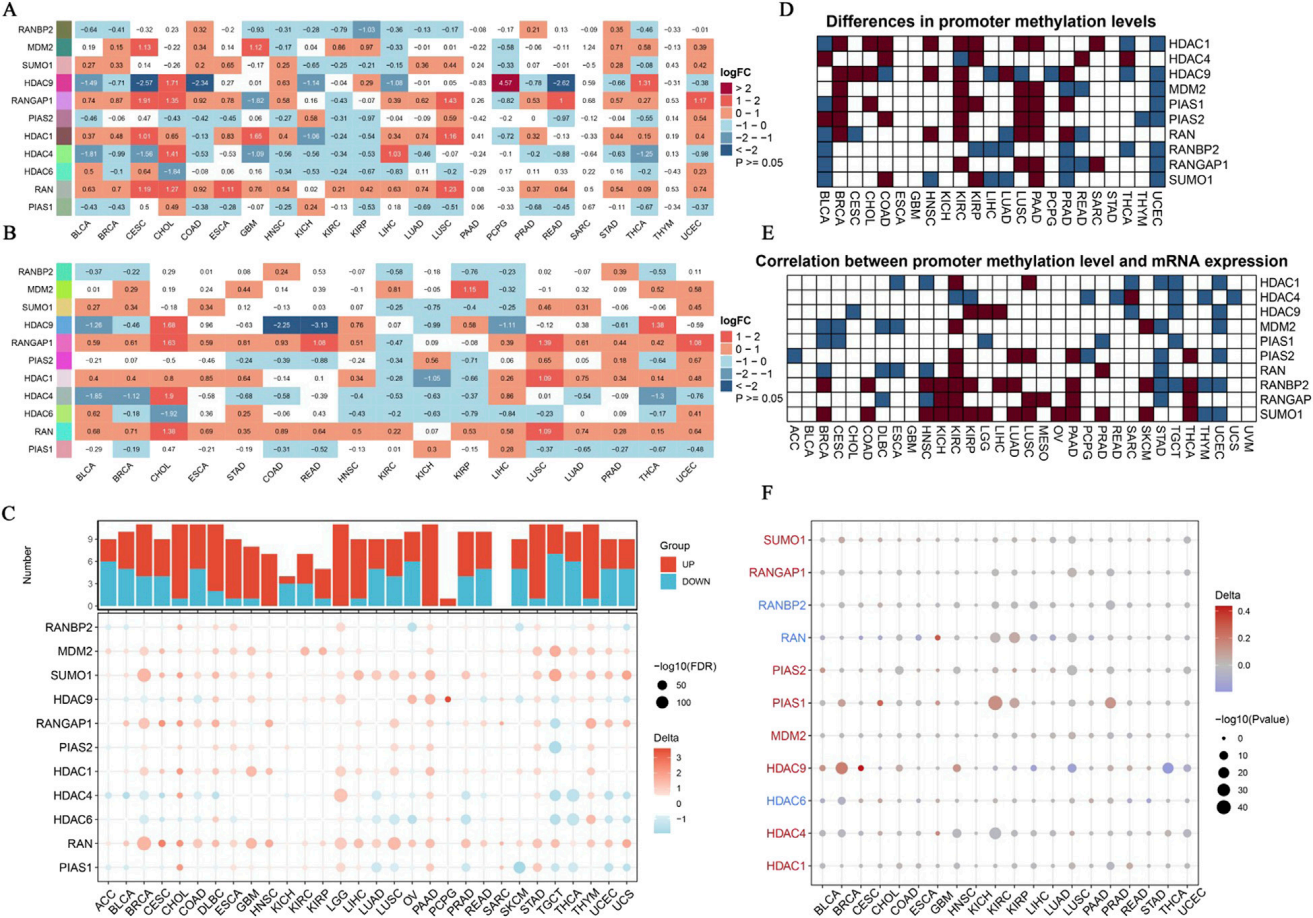
Expression Landscape of SUMOylation-Related Genes in Pan-Cancer. **(A)** Using unpaired methods, we analyzed differential gene expression driven by SUMOylation across various pan-cancer samples. Each row represents a unique SUMOylation-related gene, while each column corresponds to a specific cancer type. **(B)** This panel displays the correlation between SUMOylation-related gene expression in paired cancer samples. The heatmap, consistent with **(A)**, uses log2FC values to depict the contrast in gene expression between tumor and normal tissues within the same patients. **(C)** We explored differential expression of SUMOylation-associated genes across several datasets from TCGA-GTEx. The dot plot shows log2FC values, with dot size corresponding to the -log10 of corrected p-values. Downregulation is shown by blue dots, while upregulation is represented by red dots. **(D)** Analysis of promoter methylation in SUMOylation-related genes. The heatmap shows differences in promoter methylation levels between tumor and normal tissues, with a gradient from white to dark blue signifying increasing methylation levels. **(E)** This panel examines the correlation between promoter methylation levels and expression of SUMOylation-related genes. The heatmap displays Pearson correlation coefficients, where dark blue represents strong negative correlations, and dark red represents strong positive correlations. **(F)** Delta values showing the differences in promoter methylation levels of SUMOylation-related genes between tumor and normal tissues in pan-cancer. The bubble plot depicts delta values, with bubble size corresponding to the negative log10 of p-values, and color indicating the direction of change (red for increased methylation, blue for reduced methylation).

Our study aimed to characterize the expression patterns and promoter methylation levels of SUMOylation-related genes across diverse cancer types. The findings reveal extensive disruption and complex epigenetic regulation of these genes. Comparative analyses of gene expression in unpaired samples (Figure 2A) and paired cancer samples (Figure 2B) indicated substantial overexpression and downregulation of SUMOylation-related genes. These observations were further corroborated by an analysis using TCGA-GTEx datasets (Figure 2C), which identified significant gene expression changes across multiple datasets, reflecting widespread alterations. Promoter methylation analysis (Figure 2D) identified specific genes with marked differences in methylation levels between tumor and normal tissues, suggesting the presence of epigenetic regulatory mechanisms. Additionally, an examination of promoter methylation and gene expression (Figure 2E) revealed both positive and negative correlations, highlighting the intricate relationship between epigenetic modifications and gene expression. The identified promoter methylation variations (Figure 2F) point to genes with abnormal delta values, which could represent potential therapeutic targets. This study contributes to a deeper understanding of the molecular pathways involved in cancer progression and identifies promising biomarkers and targets for further investigation.

### SUMOylation-related gene promoter methylation analysis

This study conducted a comprehensive analysis of methylation patterns in the promoters of SUMOylation-related genes, utilizing various data types, with findings summarized in Supplementary Figure 1. The examination of the HDAC1 promoter region (Supplementary Figure 1A) shows variation in the distribution of methylated versus unmethylated sites across samples. A pie chart illustrates higher methylation levels in certain sample types, while a circular plot highlights regions of high methylation density. In the HDAC4 promoter (Supplementary Figure 1B), methylation patterns vary significantly across datasets. A bar chart presents methylation frequency, and a pie chart indicates the proportion of methylated sites. The HDAC6 promoter (Supplementary Figure 1C) analysis investigates methylation changes according to sample type, providing a detailed view of epigenetic modifications. In contrast, the HDAC9 promoter (Supplementary Figure 1D) displays distinctive methylation hotspots, which may have regulatory effects. For the MDM2 promoter (Supplementary Figure 1E), methylation levels and patterns vary depending on environmental conditions. The PIAS1 promoter (Supplementary Figure 1F) shows differential methylation across experimental groups, whereas the PIAS2 promoter (Supplementary Figure 1G) exhibits more uniform methylation patterns. The RAN promoter (Supplementary Figure 1H) indicates the percentage of methylated sites and potential regulatory impacts, with additional graphs illustrating chromatin accessibility. The RANBP2 promoter (Supplementary Figure 1I) displays some variability in methylation, which may influence gene expression. Similarly, the RANGAP1 promoter (Supplementary Figure 1J) shows notable methylation differences across samples. Finally, the SUMO1 promoter (Supplementary Figure 1K) demonstrates potential regulatory capacity over gene expression, with bar graphs displaying methylation distribution by sample type and pie charts indicating the ratio of methylated to unmethylated sites. An additional circular plot offers a comprehensive overview of the methylation landscape, displaying ATAC-seq peaks across chromosomes and providing insights into chromatin accessibility in relation to promoter methylation.

### Analysis of SUMOylation-related genes in pan-cancer: copy number variation, methylation, and tumor mutation burden

**FIGURE 3:**
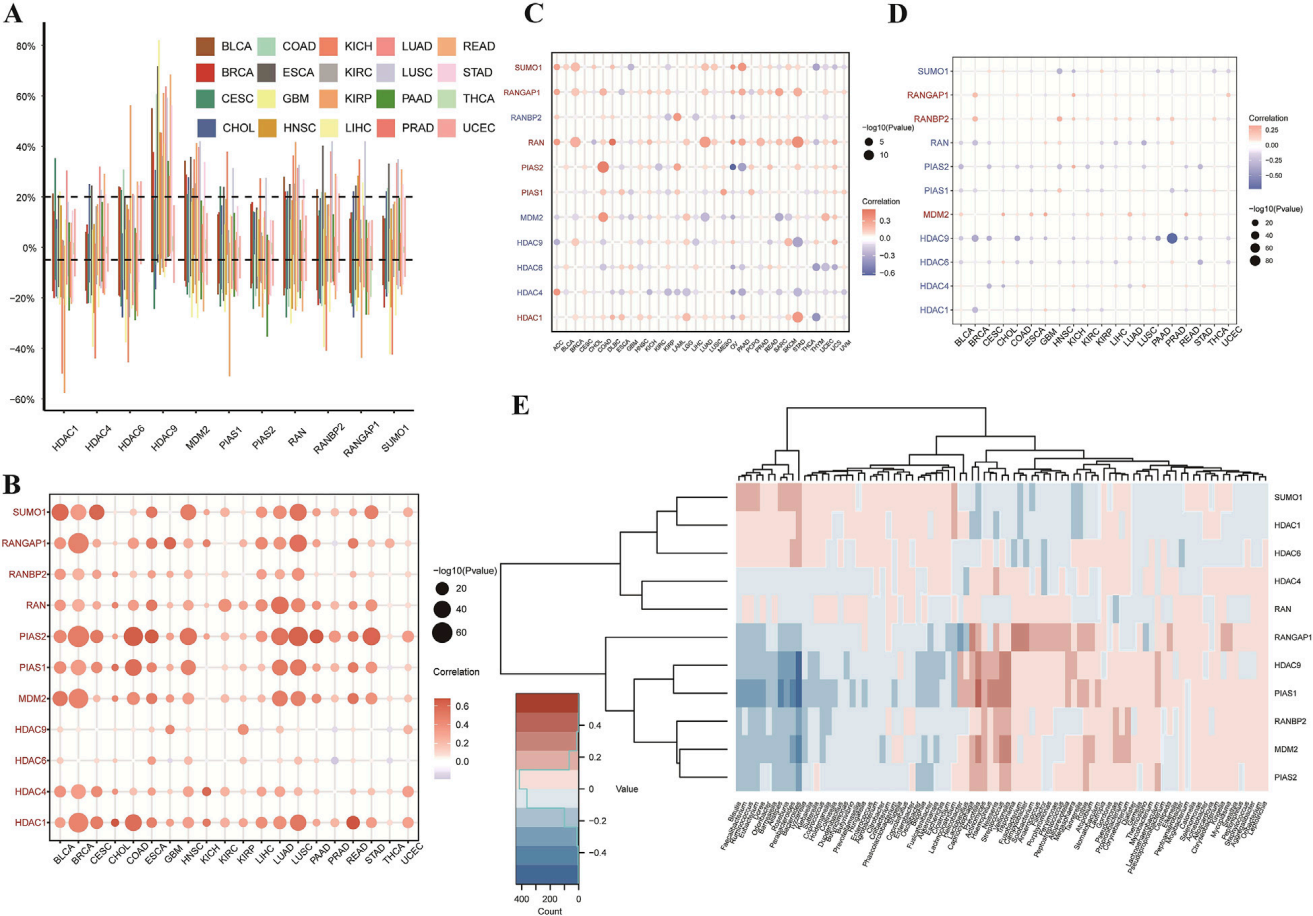
Analysis of SUMOylation-Related Genes in Pan-Cancer: Copy Number Variation, Methylation, and Tumor Mutation Burden. **(A)** The bar plot shows the rates of copy number variation (CNV) in SUMOylation-related genes across 20 different types of cancer. Each bar represents a cancer type, with colors as per the legend. Data points show variation rates, with the vertical axis displaying the percentage of samples with CNV, and the horizontal axis listing the cancer types. **(B)** Correlation of Copy Number Variation (CNV) and Gene Expression. This bubble plot illustrates the correlation between CNV and expression levels of SUMOylation-related genes across cancer types. Bubble color denotes the direction and magnitude of the correlation coefficient—red for positive correlations and blue for negative—with bubble size reflecting correlation strength. **(C)** Relationship between Tumor Mutation Burden (TMB) and Gene Expression. This bubble plot presents the association between TMB and expression of SUMOylation-related genes across various cancers. Bubble size indicates correlation significance, with color intensity showing relationship strength, similar to **(B)**. **(D)** Relationship between Promoter Methylation and Gene Expression. This bubble plot illustrates the correlation between promoter methylation and expression levels of SUMOylation-related genes across multiple cancers. Bubble size signifies correlation significance, while color indicates the direction of the relationship. **(E)** Gene Expression in Different Tumor Microenvironments. The heatmap presents expression levels of SUMOylation-related genes across various tumor microenvironments. Each row represents a gene, and each column a specific tumor type. The color gradient indicates expression levels, with pink for lower expression and blue for higher expression.

This study conducted a comprehensive analysis of SUMOylation-related genes across various cancers, focusing on genetic, epigenetic, and expression alterations. Figure 3A displays the Copy Number Variation (CNV) rates of SUMOylation-related genes across 20 cancer types, with each bar color-coded by cancer type. Figure 3B presents a bubble plot illustrating the relationship between CNV and gene expression; bubble size and color (red for positive correlation, blue for negative) indicate the strength and direction of these associations. Figures 3C, D depict similar patterns for Tumor Mutation Burden (TMB) and promoter methylation, respectively, suggesting that CNVs and hypermethylation are major drivers of abnormal gene expression in cancer. Lastly, Figure 3E showcases a heatmap representing the expression levels of SUMOylation-related genes across various tumor microenvironments, where rows correspond to genes and columns to cancer types, with a color gradient indicating expression levels (pink for lower, blue for higher). This study underscores the intricate regulatory roles of SUMOylation-related genes in cancer, offering valuable insights for developing targeted therapeutic strategies.

### Application of molecular docking and pathway enrichment analyses in low-grade glioma (LGG) and glioblastoma (GBM)

**FIGURE 4:**
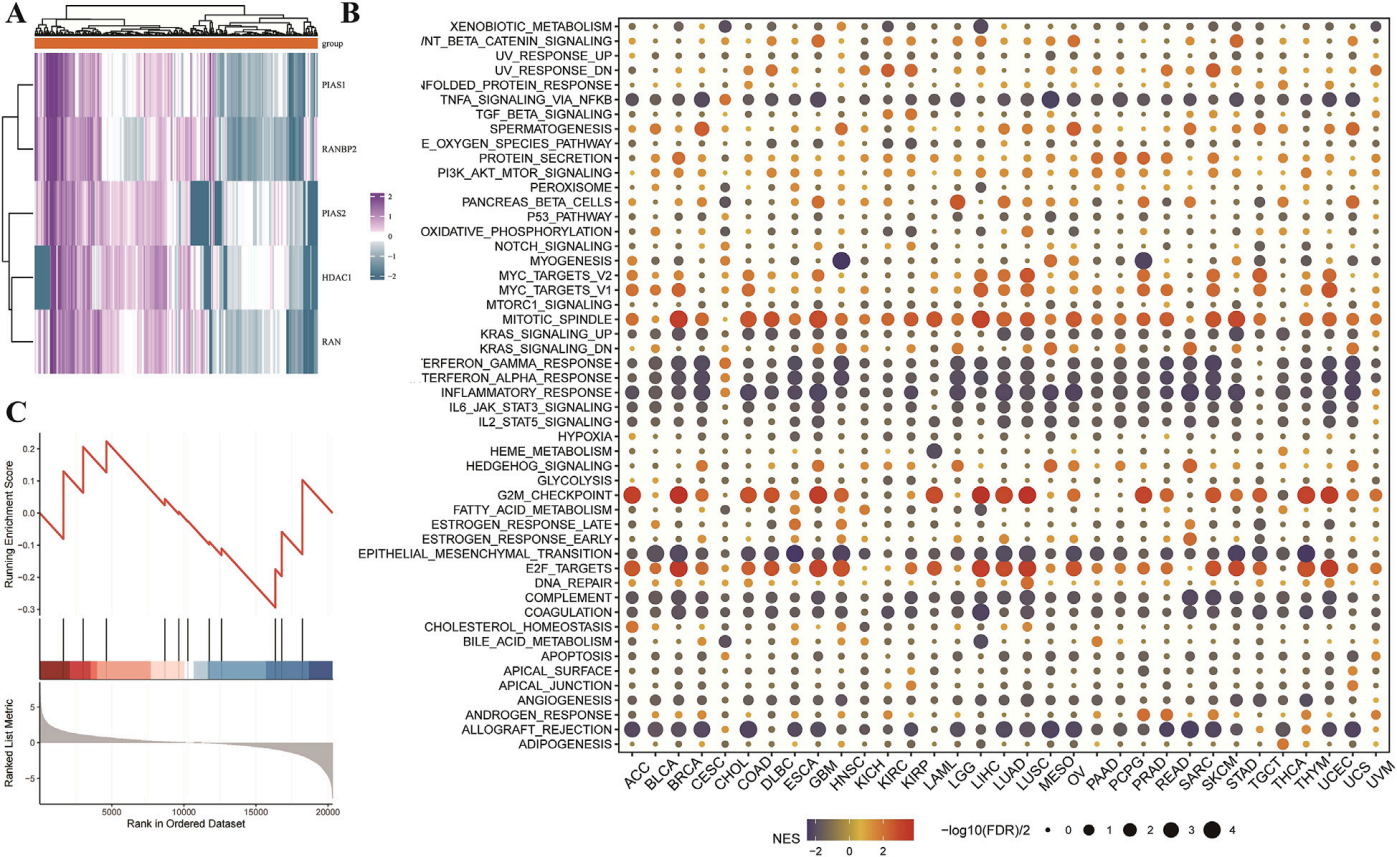
Utilizing molecular docking and pathway enrichment analyses to study Low-Grade Glioma (LGG) and GBM. **(A)** Heatmap for core protein drug sensitivity screening and molecular docking. This heatmap depicts the relationship between important proteins (HDAC1, PIAS1, PIAS2, RAN, and RANBP2) and the prognosis of low-grade glioma (LGG) patients, as determined by a pan-cancer investigation. These proteins have been identified as important predictors of poor prognosis in LGG. A simulated molecular docking research was performed to discover possible therapeutic medicines that target these proteins. These major proteins’ three-dimensional structures were acquired from the PDB database, and 321 small molecule ligands were identified from the NCBI PubChem database. These ligands were then molecular docked with the target proteins to determine their binding affinities, which were calculated using LibDockScore. The data show that Dfo, Paromomycin, and 5-Methyltetrahydrofolate have strong binding affinities to HDAC1, PIAS1, PIAS2, RAN, and RANBP2, implying that they might be used as therapeutic candidates to target ubiquitin-like modification pathways in LGG. **(B)** Pan-cancer. GSEA enrichment analysis: The dot plot depicts the enrichment analysis of gene sets connected to distinct signaling pathways across cancer types using the GSEA approach. The normalized enrichment score (NES) is shown by color gradients, with red indicating a positive NES (enriched in the cancer group) and blue indicating a negative NES (enriched in the control group). The dots’ sizes show the enrichment’s significance level (-log10(FDR q-value)) for each gene set. Significant processes that have been enhanced include xenobiotic metabolism, epithelial-mesenchymal transition, and fatty acid metabolism. **(C)** GSEA enrichment analysis of the sumoylation-related gene sets in GBM: The figure depicts the enrichment analysis of sumoylation-related gene sets in GBM versus normal tissues, which was performed using the clusterProfiler software. The enrichment score curve shows the ranking of genes according on their expression levels in the GBM and control groups. The bar plot under the curve shows the position of genes in the ranked list, demonstrating the degree of enrichment for sumoylation-related activities in GBM.

This research also performed a detailed analysis to identify critical proteins associated with LGG prognosis. Results indicate that HDAC1, PIAS1, PIAS2, RAN, and RANBP2 are significant markers of poor prognosis in LGG. These proteins were subsequently analyzed through molecular docking to identify potential therapeutic candidates. Three-dimensional structures of these proteins were retrieved from the PDB database, and 321 small chemical ligands were sourced from the NCBI PubChem database for screening. Results indicated that Dfo, Paromomycin, and 5-Methyltetrahydrofolate exhibited strong binding affinities to HDAC1, PIAS1, PIAS2, RAN, and RANBP2, suggesting their potential as therapeutic candidates targeting ubiquitin-like modifications in LGG (Figure 4B). Gene set enrichment analysis (GSEA) was performed across multiple cancer types to identify critical pathways involved in cancer progression (Figure 4B). In Figure 4B, a dot plot presents the normalized enrichment scores (NES) for multiple gene sets, with dot size reflecting the enrichment significance of each gene set. Key pathways such as xenobiotic metabolism, epithelial-mesenchymal transition (EMT), and fatty acid metabolism were prominently enhanced in cancer groups. Additionally, GSEA was applied to examine molecular processes in GBM, focusing on SUMOylation-related gene sets. Figure 4C shows an enrichment score curve, ranking genes by expression levels in GBM versus control groups. The histogram above the curve illustrates gene positions in the ranked list, indicating their association with SUMOylation-related functions in GBM. Findings revealed higher enrichment of SUMOylation-related gene sets in GBM than in normal tissues, emphasizing SUMOylation’s role in GBM tumorigenesis. The comprehensive analyses presented here provide new insights into the molecular mechanisms and potential therapeutic pathways for LGG and GBM, mediated through cancer-specific protein interactions and reconfiguration of signaling networks.

### Assessing the prognostic relevance of SUMOylation-related genes in glioblastoma via single-sample gene set enrichment analysis (ssGSEA)

**FIGURE 5:**
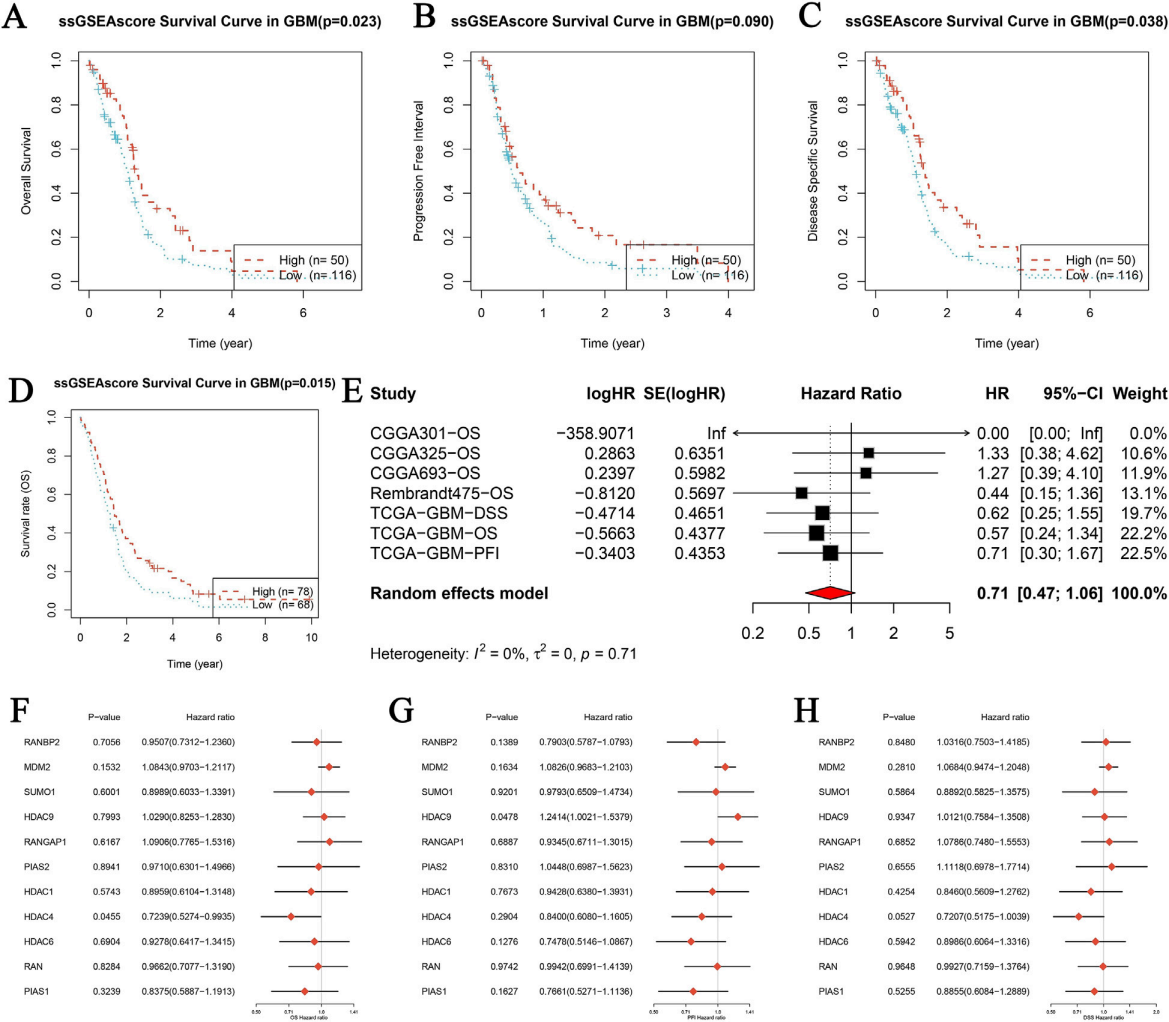
Prognostic analysis of genes related to SUMOylation in glioblastoma (GBM) using single-sample gene set enrichment analysis (ssGSEA). **(A–D)** Kaplan-Meier survival analysis was performed for three distinct survival outcomes in GBM: Overall Survival (OS) **(A)**, Progression-Free Interval (PFI) **(B)**, and Disease-Specific Survival (DSS) **(C)**. The study contrasts high and low ssGSEA scores for SUMOylation-related gene expression, with p-values indicating statistical significance. The datasets utilized include publicly accessible GBM patient data. **(E)** Meta-analysis of univariate Cox proportional hazards regression across several datasets for overall survival (OS) in GBM. The analysis incorporates papers from CGGA301, CGGA325, CGGA693, Rembrandt, and TCGA. The forest plot displays logHR, SE (logHR), Hazard Ratio (HR), 95% Confidence Interval (CI), and weight for each research. The random-effects model calculates the combined hazard ratio using heterogeneity statistics. **(F–H)** Forest plots of the hazard ratios for individual SUMOylation-related genes across various GBM datasets for OS **(F)**, PFI **(G)**, and DSS **(H)**. Each figure displays the p-value, hazard ratio, and confidence intervals for the genes studied, providing information on their prognostic relevance.

Kaplan-Meier survival analyses were conducted to assess the prognostic significance of SUMOylation-related genes in glioblastoma (GBM). Scores derived from Single-Sample Gene Set Enrichment Analysis (ssGSEA) were used to evaluate three survival outcomes: Overall Survival (OS), Progression-Free Interval (PFI), and Disease-Specific Survival (DSS). Kaplan-Meier curves revealed significant differences in survival rates between the high and low ssGSEA score groups. Specifically, higher ssGSEA scores correlated with worse overall survival (Figure 5A, p = 0.022), shorter progression-free intervals (Figure 5B, p < 0.001), and reduced disease-specific survival (Figure 5C, p = 0.038). Further analysis of OS confirmed these findings (Figure 5D, p = 0.015). To strengthen these observations, a combined analysis of multiple GBM datasets (CGGA301, CGGA325, CGGA693, Rembrandt, and TCGA) was performed using univariate Cox proportional hazards regression. This analysis indicated that higher ssGSEA scores were associated with decreased survival, with a combined hazard ratio (HR) of 0.71 (95% CI: 0.47–1.06), reflecting a reduced risk of death. There was no significant variability in results across the datasets (Figure 5E). Additionally, analysis of individual genes provided further insights into the prognostic significance of SUMOylation-related genes for OS (Figure 5F), PFI (Figure 5G), and DSS (Figure 5H). These findings collectively suggest that elevated ssGSEA scores of SUMOylation-related genes are associated with poorer prognostic outcomes in GBM patients, highlighting their potential as valuable prognostic biomarkers.

### Prognostic model of SUMOylation-related genes in GBM

The primary objective of this study was to develop and validate a predictive model based on the expression of SUMOylation-related genes in GBM. Supplementary Figure 2A displays the calibration curve and goodness-of-fit test for the ssGSEA score in distinguishing between tumor and normal groups. The red line represents the observed findings, while the dashed blue line signifies the ideal prediction. The Hosmer-Lemeshow test indicated satisfactory concordance between observed and predicted probabilities. In Supplementary Figure 2B, a comparison of ssGSEA scores between tumor and normal groups reveals no significant distinction, indicating that ssGSEA scores do not substantially differ between tumor and normal tissues in GBM. The diagnostic performance of the ssGSEA score for differentiating between tumor and normal groups was evaluated using a ROC curve (Supplementary Figure 2C), which suggested limited diagnostic capability. These findings indicate that while the ssGSEA score model is well-calibrated, its ability to distinguish between tumor and normal tissues in GBM is limited, as evidenced by the minimal difference in ssGSEA scores and the modest AUC value. Further research is warranted to enhance the model’s accuracy and improve its diagnostic utility for GBM.

### Paromomycin suppresses the activity of genes involved in SUMOylation modification and decreases the viability of GBM cells

**FIGURE 6:**
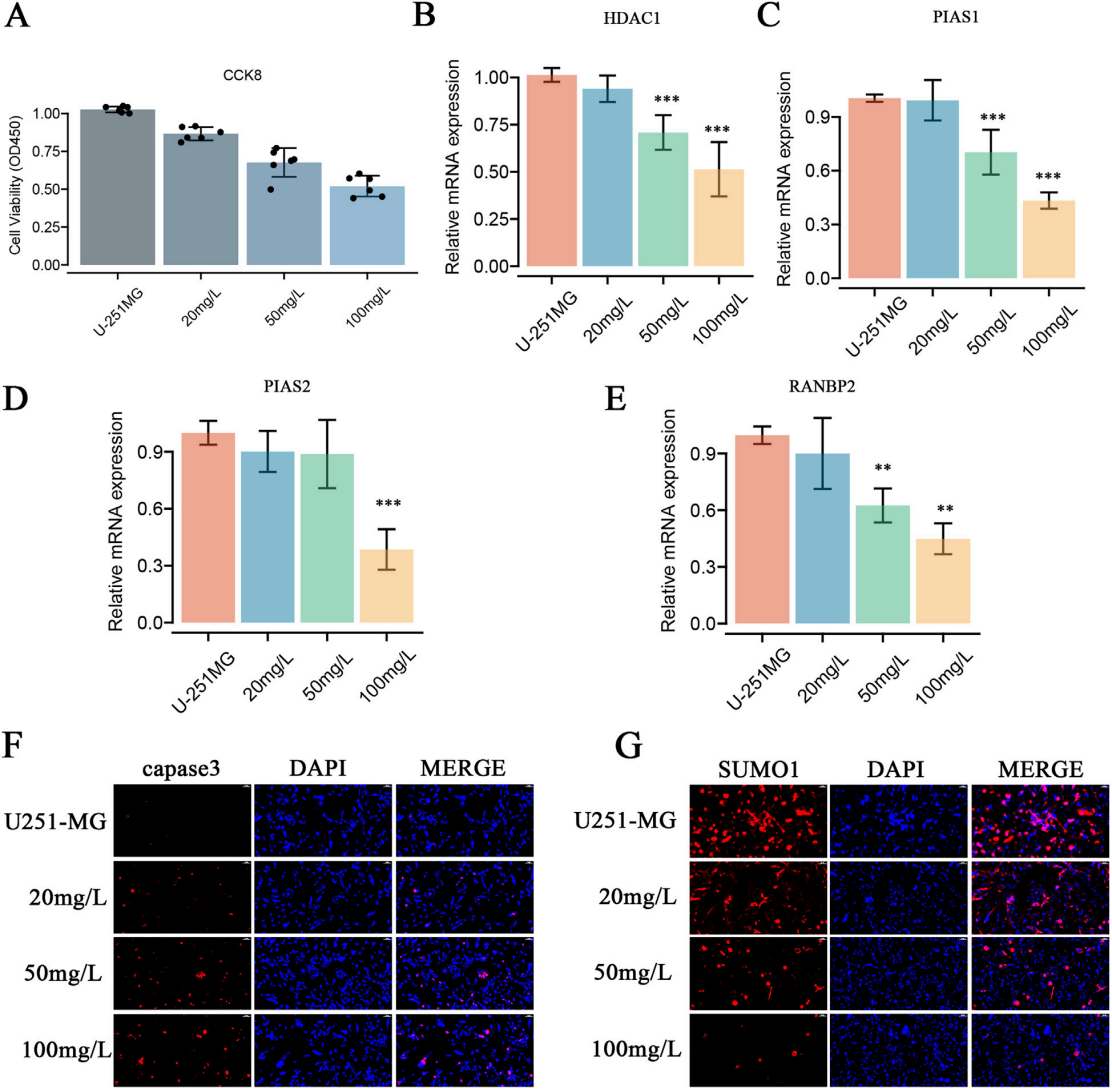
Effects of Paromomycin on Cell Viability, Gene Expression, Apoptosis, SUMOylation, and Colony Formation in U-251MG Glioblastoma Cells. **(A)** Cell viability was assessed using the CCK8 assay. U-251MG cells were treated with varying concentrations of Paromomycin (20 mg/L, 50 mg/L, 100 mg/L), and the optical density (OD450) was measured. The results show a dose-dependent decrease in cell viability, indicating that Paromomycin effectively reduces the proliferation of U-251MG cells. **(B–E)** qRT-PCR analysis of relative mRNA expression levels of HDAC1, PIAS1, PIAS2, and RANBP2 after treatment with Paromomycin at different concentrations. The data show a significant downregulation of these genes in a dose-dependent manner, with the highest inhibition observed at 100 mg/L. Statistical significance was indicated as follows: **p < 0.01, ***p < 0.001 compared to the untreated control group. **(F)** Immunofluorescence staining for caspase-3 (red), a key marker of apoptosis, in U-251MG cells treated with increasing concentrations of Paromomycin. The results showed increased caspase-3 expression, indicating that apoptosis was induced by Paromomycin in U-251MG cells. **(G)** Immunofluorescence staining was performed to assess the levels of the SUMOylation protein (SUMO1, shown in red). Nuclei are stained with DAPI (blue). The results indicate that Paromomycin actively inhibits protein SUMOylation, as evidenced by a significant reduction in SUMO1 expression across various drug concentrations.

**FIGURE 7:**
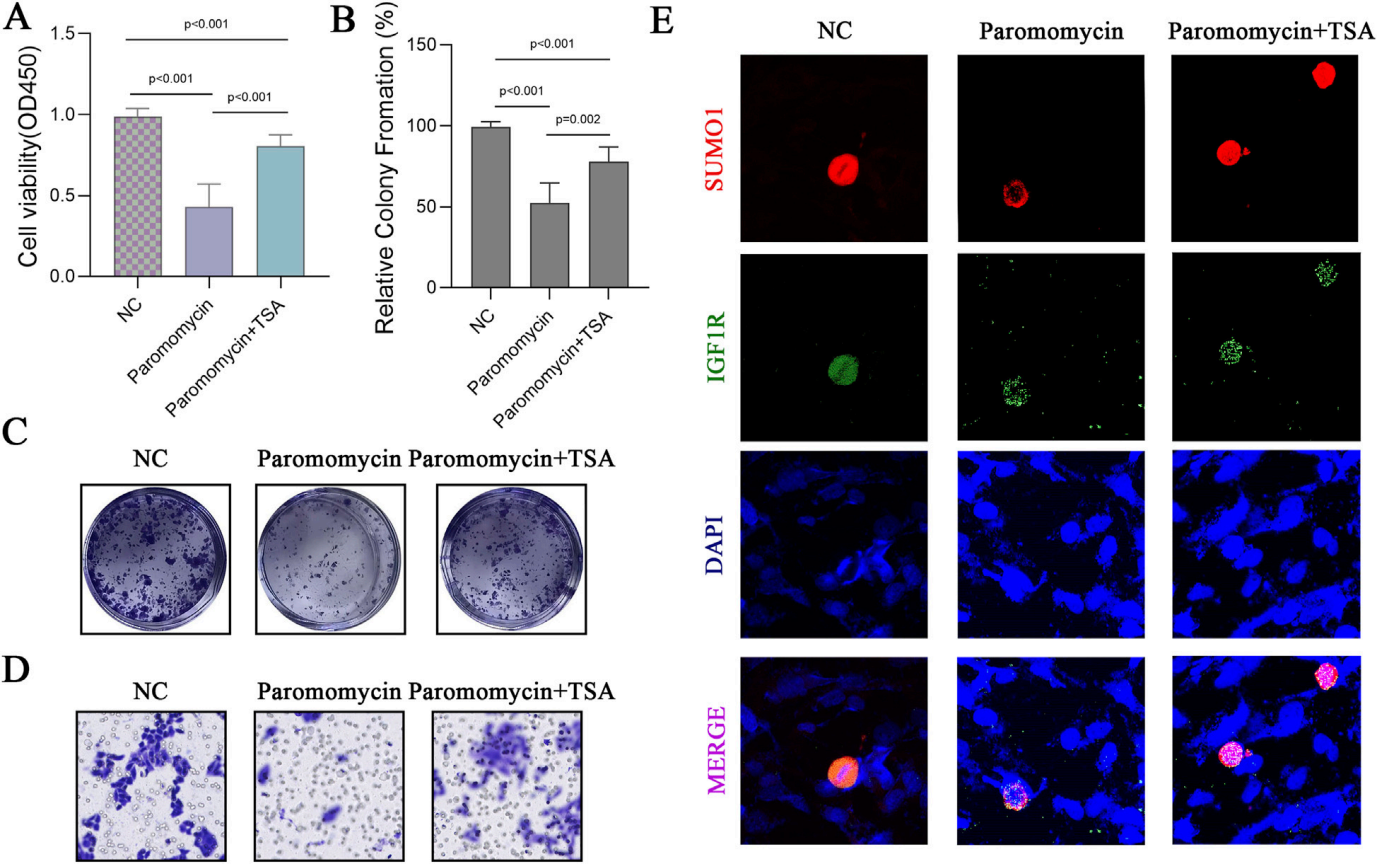
Effects of Paromomycin on Cell Viability, Colony Formation, Migration, and SUMOylation in U-251MG Glioblastoma Cells. **(A)** U-251MG glioblastoma cells were treated with Paromomycin and Paromomycin + TSA, and cell viability was assessed using the CCK8 assay. Optical density (OD450) values indicate a significant reduction in cell viability in the Paromomycin-treated group compared to the NC (negative control), with further reduction observed when combined with TSA. Statistical significance levels are indicated (*p < 0.001). **(B)** Quantitative analysis of colony formation assay, presented as relative colony formation percentages. Paromomycin treatment alone and in combination with TSA significantly decreased colony formation compared to the NC group. Statistical significance levels are indicated (*p < 0.001, **p = 0.002). **(C)** Representative images from the colony formation assay in U-251MG cells. Paromomycin treatment reduced both colony number and size, with an enhanced effect in combination with TSA. **(D)** Representative images from the transwell migration assay for U-251MG cells under the NC, Paromomycin, and Paromomycin + TSA conditions. **(E)** Immunofluorescence staining in U-251MG cells to assess the effect of Paromomycin on SUMO1 and IGF1R nuclear translocation. Red indicates SUMO1 staining, green indicates IGF1R, and blue represents DAPI-stained nuclei. The images suggest that Paromomycin reduces IGF1R nuclear translocation, possibly associated with SUMO1 modification.

**FIGURE 8:**
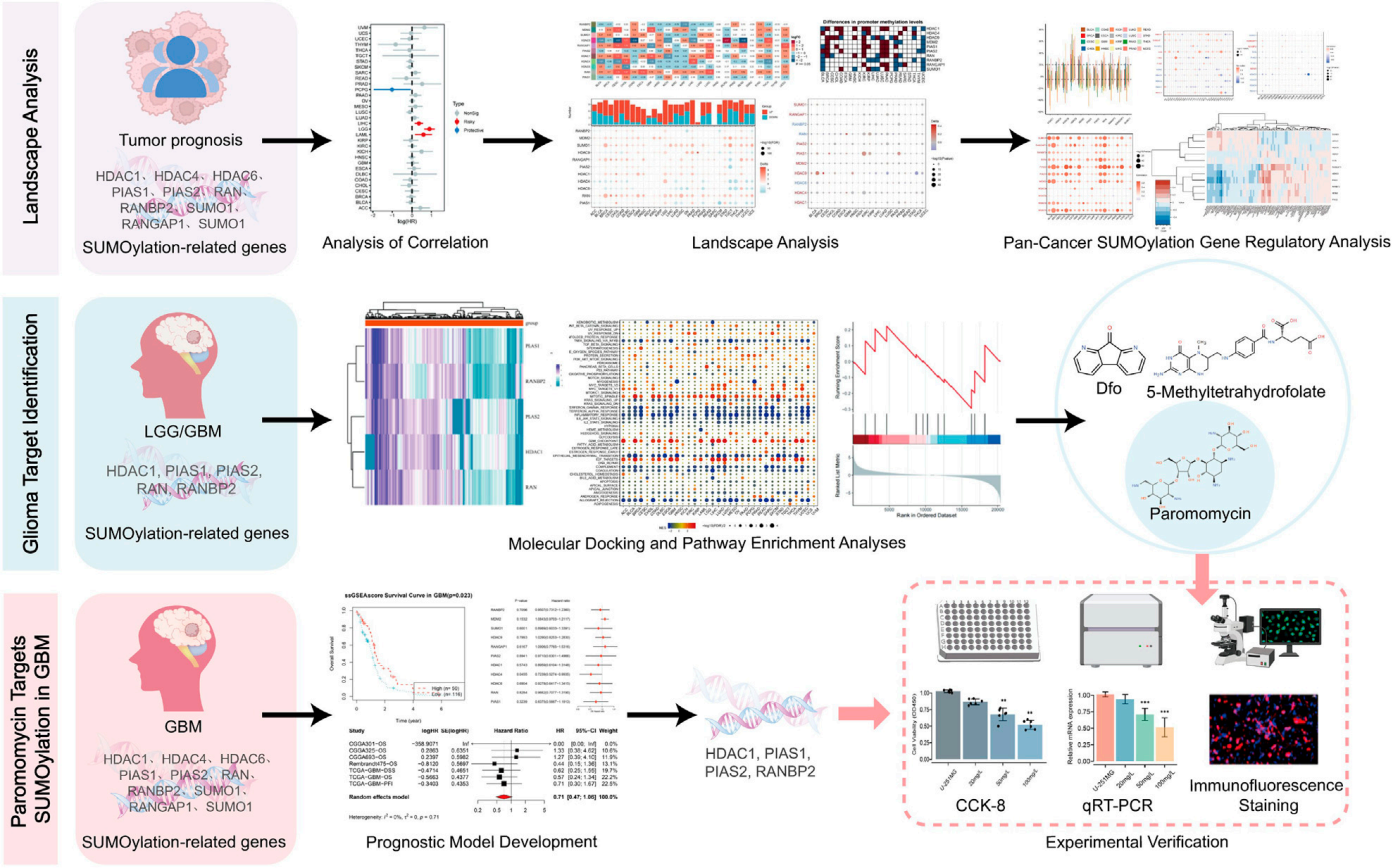
Integrated bioinformatics and experimental analysis of Paromomycin targeting HDAC1 in GBM.

Paromomycin suppresses the activity of SUMOylation-related genes and decreases the viability of GBM cells. Our study explored the effects of Paromomycin on GBM cell survival and SUMOylation gene expression. Figure 6A illustrates that Paromomycin treatment led to a dose-dependent reduction in cell viability. Additionally, qRT-PCR analysis revealed significant reductions in the mRNA levels of HDAC1, PIAS1, PIAS2, and RANBP2 in Paromomycin-treated GBM cells as the dosage increased (Figures 6B–E). Immunofluorescence labeling of caspase-3 and SUMO1 further demonstrated that higher doses of Paromomycin enhanced caspase-3 fluorescence intensity while decreasing SUMO1 expression (Figures 6F, G). A colony formation assay also indicated that Paromomycin reduced glioma cell proliferation (Supplementary Figures 2D, E). In U-251MG glioblastoma cells, Paromomycin treatment significantly decreased cell viability, as shown in Figure 7A. The CCK8 cell proliferation assay demonstrated a dose-dependent reduction in the OD450 value in Paromomycin-treated cells compared to the negative control (NC), with a greater reduction observed when Paromomycin was combined with TSA. This finding suggests that Paromomycin has a potent antiproliferative effect, particularly in combination with TSA. The colony formation assay further validated the inhibitory effect of Paromomycin on cell proliferation (Figures 7B, C). Quantitative analysis showed a marked reduction in colony formation in the Paromomycin group relative to the NC, while the addition of the HDAC1 inhibitor TSA increased colony formation. In a transwell migration assay (Figure 7D), Paromomycin was found to impair U-251MG cell migration in addition to reducing cell viability and proliferation. The images showed a substantial reduction in migrating cells following Paromomycin treatment, an effect that was reversed with TSA co-treatment. Immunofluorescence analysis provided insight into Paromomycin’s molecular action, particularly its influence on SUMO1 modification and IGF1R nuclear translocation (Figure 7E). Paromomycin treatment decreased IGF1R nuclear translocation, possibly due to alterations in SUMO1 modification, an effect reversed by TSA treatment. These results imply that SUMO1 modification might contribute to Paromomycin’s antitumor effects, potentially involving HDAC1 regulation. Collectively, these findings suggest that Paromomycin not only diminishes GBM cell survival but also modulates the expression of critical SUMOylation-related genes, highlighting its potential as a therapeutic agent for GBM treatment (Figure 8).

## Discussion

GBM is a very aggressive and fatal brain tumor with a poor prognosis and a significant recurrence risk. Despite breakthroughs in research and treatment, the five-year survival rate for GBM patients remains less than 5%, emphasizing the critical need for new therapeutic techniques (Aldoghachi et al., 2022; Stylli, 2020). Our findings show that Paromomycin, an aminoglycoside antibiotic, has the ability to target and regulate HDAC1 and hence prevent GBM growth. Recent advancements in technology and molecular research have significantly enhanced our understanding of diseases, thereby supporting therapeutic methodologies (Liu and Ren, 2023; Candelli and Franceschi, 2023; Ciurea et al., 2023). Intensive investigations into gene expression and regulatory mechanisms within biological contexts have provided valuable insights into gene functionality (Qin et al., 2024; Ren et al., 2023; Zhao et al., 2024). Researchers have frequently highlighted the critical roles of protein-protein interaction networks and their regulatory variations in biological systems, emphasizing their significance in cell signal transduction and functional control (Tian et al., 2023; Liu et al., 2023; Zhong et al., 2019). These studies not only deepen our comprehension of disease processes but also offer robust theoretical and experimental support for future treatment modalities (Du and Liu, 2024; Chen Y-C. et al., 2024; Zeng et al., 2024; Kong et al., 2024; Di Bonito et al., 2024; Fareed et al., 2024).

The integration of biomarkers utilizing big data and bioinformatics is increasingly pivotal for disease diagnosis and predicting future health conditions (Liang et al., 2024; Yao et al., 2024; Gao et al., 2024; Zhang et al., 2024). The recognition of cell death mechanisms and metabolic control advances has introduced new therapeutic targets and perspectives in drug research (Wan et al., 2024; Yang et al., 2023; Dong et al., 2024; Ahmed et al., 2024). Understanding cytokine functions in immune responses has yielded valuable insights for developing effective treatment strategies for numerous diseases (Sheng et al., 2024). Furthermore, exercise-related studies on monocyte gene expression regulation in Alzheimer’s patients may unveil potential therapeutic pathways (Huang J. et al., 2022; Wang et al., 2020; Sun et al., 2022). Our findings reveal a relationship between elevated HDAC1 expression and poor prognoses across multiple cancer types, suggesting that HDAC1 could serve as both a prognostic marker and a therapeutic target. This study explores the role of Paromomycin in selectively targeting and modulating HDAC1 to inhibit GBM growth, utilizing bioinformatics analysis to validate experimental results. Analysis of genes associated with SUMO modification across diverse malignancies revealed significant variations in gene expression and distinct methylation patterns.

HDAC1 is a crucial enzyme involved in chromatin remodeling, leading to the repression of genetic information through the removal of acetyl groups from histone proteins (Seto and Yoshida, 2014). Additionally, HDAC1 belongs to a broader family of histone deacetylases, known for their critical roles in cell cycle progression, differentiation, and apoptosis regulation (Meunier et al., 2006). Dysregulation or dysfunction of HDAC1 is associated with the development of various malignancies, including GBM. High levels of HDAC1 in GBM significantly contribute to aggressive tumor behavior, enhancing cell proliferation, migration, and resistance to apoptosis. The overexpression of HDAC1 correlates with the inhibition of tumor suppressor genes and activation of oncogenic pathways, thereby promoting tumor growth and survival. Moreover, HDAC1’s role in maintaining the self-renewal potential of GBM cancer stem cells complicates treatment efforts, as these cells exhibit resistance to standard therapies (Lo et al., 2021). Given HDAC1’s substantial involvement in GBM progression, it presents a promising therapeutic target. Our study demonstrates that Paromomycin acts as an HDAC1 inhibitor, reversing detrimental epigenetic modifications that fuel tumor growth and survival. Such drugs can activate genes that restrict tumor proliferation or enhance apoptotic pathways, thereby increasing the efficacy of conventional therapies.

The development of innovative targeted therapies has the potential to improve treatment effectiveness while minimizing adverse side effects (Vargas-Sierra et al., 2024; Wahi et al., 2024; Ma et al., 2024), thereby advancing precision medicine. Paromomycin, an aminoglycoside antibiotic, has been minimally investigated for its anticancer potential (Hirukawa et al., 2005). Through molecular docking studies, we identified Paromomycin from an FDA-approved drug library as a promising HDAC1 inhibitor. Its strong binding affinity to HDAC1 indicates that Paromomycin can regulate HDAC1 activity and influence the SUMOylation pathway, representing a novel approach to specifically target this pathway in GBM and potentially yield new treatment options. Our research suggests that Paromomycin may effectively inhibit GBM by targeting HDAC1 to regulate IGF1R SUMOylation, potentially opening new avenues for GBM treatment and inspiring exploration of similar mechanisms in other cancer types (Kunadis et al., 2021). HDAC1 (histone deacetylase 1) is known to promote tumor growth in various cancers by regulating chromatin structure through deacetylation, thus influencing gene expression (Olzscha et al., 2015). In GBM, abnormal HDAC1 activity is closely associated with increased cell proliferation, invasiveness, and poor prognosis. Therefore, inhibiting HDAC1 with Paromomycin may disrupt key signaling pathways controlled by HDAC1, potentially reducing tumor cell proliferation and invasiveness. In laboratory assays, we confirmed that Paromomycin interacts specifically with and modulates SUMOylated HDAC1 protein, resulting in a significant reduction in GBM cell growth, motility, and invasiveness (Fox et al., 2019; Cheng et al., 2023; Guo et al., 2022). *In vitro* experiments demonstrated that Paromomycin effectively inhibits the proliferation of U-251MG, a widely used GBM cell line, with the CCK-8 proliferation assay indicating a clear decrease in cell viability in relation to Paromomycin concentration. Additionally, qRT-PCR analyses revealed decreased expression of HDAC1 and other SUMO-related genes, such as PIAS1, PIAS2, and RANBP2, in the presence of Paromomycin. Immunofluorescence staining corroborated these findings, showing increased levels of caspase-3 and decreased levels of SUMO1, indicative of enhanced apoptotic activity and reduced protein SUMOylation.

In recent years, the development of novel targeted therapies has not only enhanced treatment efficacy and reduced adverse effects but also promoted advancements in precision medicine (Vargas-Sierra et al., 2024). Systematic reviews and meta-analyses have been widely applied in biomedical research, covering various methodological studies such as drug development and bioinformatics, significantly advancing both basic research and translational medicine (Wu Z. et al., 2024). Additionally, the pivotal role of cell death and metabolic regulation in disease progression has gained increasing attention, providing new targets for drug research (Lin et al., 2023). New drugs targeting specific proteins or gene pathways have notably improved the specificity and effectiveness of treatments (Hong et al., 2024). For instance, research has shown that kiwi root extract exerts therapeutic effects on gastric cancer (Chu et al., 2023). The integration of modern technology with traditional Chinese medicine also provides new perspectives and potential in drug development (Wang et al., 2023). Advances in materials science have led to the development and application of various novel composite materials, showing broad potential in biomedical and engineering fields (Wu H. et al., 2024). Paromomycin, an aminoglycoside antibiotic, is commonly used to treat intestinal infections and amebiasis (Botero, 1970). Recent studies have begun exploring its potential in cancer therapy, particularly its impact on histone deacetylase 1 (HDAC1). Research suggests that Paromomycin may inhibit HDAC1 activity, altering the epigenetic state of tumor cells and thereby reducing their proliferation and invasiveness. The insulin-like growth factor 1 receptor (IGF1R) plays a crucial role in cell proliferation and survival, with its nuclear localization closely associated with the regulation of specific gene expression. Given Paromomycin’s established use, known safety profile, and pharmacokinetics, repurposing it as an HDAC1-targeted anticancer drug could potentially lower the costs and risks associated with new drug development.

In tumors and neurological disorders, SUMOylation regulation of specific receptors or proteins can significantly impact key processes such as cell proliferation, differentiation, and apoptosis (Liu et al., 2017). IGF1R, a transmembrane receptor tyrosine kinase, is heavily involved in cell proliferation, differentiation, survival, and metabolic regulation (Romano, 2003). Additionally, IGF1R plays a crucial role in nervous system development, neuron survival, and synaptic plasticity, making its functional regulation closely tied to the progression of various diseases (Dyer et al., 2016). SUMOylation of IGF1R can alter downstream signal transmission efficiency, thus inhibiting tumor cell proliferation and migration (Zhang et al., 2015). By specifically modulating IGF1R SUMOylation, targeted therapeutic strategies could be designed that differ from traditional IGF1R inhibitors, potentially reducing side effects and enhancing therapeutic efficacy (Zhang et al., 2015; Chen et al., 2024b). Research indicates that HDAC1 is involved not only in acetylation regulation but also interacts with other post-translational modifications, such as SUMOylation. HDAC1 activity may influence the expression or function of SUMO-related enzymes, affecting the SUMOylation status of key proteins like IGF1R. Thus, HDAC1 inhibitors, such as Paromomycin, could regulate IGF1R’s signaling function and mechanisms in tumors by altering its SUMOylation level. This regulatory approach may aid in understanding the complex interactions between HDAC1 and SUMOylation in tumorigenesis and tumor progression. Targeting HDAC1 to modulate IGF1R SUMOylation holds significant implications for cancer research and therapy. This study not only helps to deepen the understanding of the molecular mechanisms of tumorigenesis but may also open new therapeutic avenues, offering more effective treatment options and solutions to overcome cancer drug resistance. These findings may have profound impacts on multiple tumor types, including GBM, in the future. Our findings suggest that HDAC1 activity may affect IGF1R nuclear translocation. Specifically, inhibition of HDAC1 could lead to a reduction in IGF1R SUMO1 modification, altering its nuclear localization and, consequently, downstream signaling and gene expression.

Furthermore, although results indicate no significant correlation between HDAC1 levels and overall survival in GBM, nor substantial differences in methylation levels, this does not directly negate the role of SUMOylation in GBM. SUMOylation is a complex post-translational modification that can influence tumor growth and progression through various mechanisms. While the direct association between HDAC1 and other SUMO-related genes with GBM was not prominent in the current data, this may be due to their indirect or context-specific roles in tumor biology. For instance, the regulation of HDAC1 activity may alter IGF1R SUMOylation levels, subsequently impacting cellular signaling, proliferation, and invasive capabilities. This effect may emerge under specific experimental conditions but could be diluted by heterogeneity in large clinical datasets. SUMOylation involves multiple genes and proteins, which may display heterogeneous effects across different cell types or tumor stages. Although statistical significance for SUMO-related genes in GBM is not strong in this study, this does not exclude the potential impact of unexamined SUMO genes or subgroups. SUMOylation can modulate tumor-suppressing and tumor-promoting genes, such as the enhanced function of the oncogenic protein MDM2 upon SUMOylation, which promotes cell proliferation and survival, driving tumor progression (Chen et al., 2025). This mechanism is especially relevant in glioma and other tumors, where SUMOylation regulation could influence treatment outcomes. SUMOylation also plays a crucial role in protein nuclear translocation (Chen et al., 2024b; Chen et al., 2024c). Therefore, deeper exploration of specific SUMO-related gene functions or differential effects across tumor subtypes may reveal new insights.

The impact of exercise on nuclear translocation has been studied across various biological pathways, particularly in metabolic regulation and cell signaling (McGee et al., 2003). Exercise is believed to slow tumor progression through various mechanisms, including immune modulation, reducing pro-inflammatory factors, and improving metabolic status. The exercise-regulated nuclear translocation mechanism is essential for cell repair, adaptation, and antioxidant responses. If Paromomycin can influence specific nuclear translocation pathways through HDAC1, it may enhance exercise’s biological effects. This could be particularly beneficial for older adults or patients with chronic illnesses, where exercise-induced metabolic adaptation is limited, providing extra metabolic support to improve exercise outcomes. As an HDAC1 inhibitor, Paromomycin may impact gene expression, metabolic responses, and anti-tumor activity related to exercise adaptation. However, experimental validation is needed to confirm its specific effects on nuclear translocation and its biological impact.

In summary, personalized medicine, targeted therapy, and immunotherapy are actively under investigation to enhance patient quality of life and treatment efficacy (Yang and Cai, 2023; Jackson and Chester, 2015). Scientists are continuously investigating and developing new therapeutic strategies by combining several research approaches including machine learning and bioinformatics technologies, including small molecule compound screening, multi-omics analysis, deep learning, and bioinformatics techniques, so offering new possibilities for precision medicine and personalized treatment (Wahi et al., 2024; Li et al., 2024; Lan et al., 2024; Yin et al., 2024). It is recommended to use shared decision-making components and checklists in clinical practice to improve patient involvement and satisfaction, especially when it comes to choosing drugs and planning therapy (Li et al., 2023; Shan et al., 2024). Advancements in drug delivery systems and the application of nanotechnology have the potential to greatly improve medication targeting and boost therapeutic effectiveness. Co-administration of Paromomycin with other therapeutic agents is also proposed to enhance its effectiveness, contributing to a comprehensive strategy against GBM. This work lays the groundwork for future exploration and drug development aimed at translating these findings into clinically effective therapies for GBM (Vargas-Sierra et al., 2024; Wu et al., 2023; Abuaisheh and Aboud, 2023; Cui et al., 2023; Guo Q. et al., 2023). Further studies are necessary to elucidate the specific molecular mechanisms through which Paromomycin affects HDAC1 and the SUMOylation pathway. It is essential to assess the gene expression levels and methylation status of SUMOylation-related genes in human GBM samples, ensuring that our findings are applicable to clinical scenarios.

## Conclusion

This study highlights the critical role of SUMOylation-related genes in cancer prognosis. Paromomycin shows potential for treating GBM by reducing cell viability and migration and impacting SUMO1 modification and IGF1R nuclear translocation. These findings suggest that targeting SUMOylation-related pathways with Paromomycin may offer a promising strategy for GBM treatment, paving the way for new targeted therapies in cancer.

## Data Availability

The original contributions presented in the study are included in the article/Supplementary Material, further inquiries can be directed to the corresponding author.

## Supplementary material

SUPPLEMENTARY FIGURE 1 Promoter Methylation Analysis of SUMOylation-Related Genes. This diagram illustrates the methylation levels of the promoters for various SUMOylation-related genes across multiple datasets. **(A)** HDAC1 Promoter Methylation Analysis: This panel depicts the methylation status of the HDAC1 promoter region, including both the methylation rate and the unmethylated rate in different samples. **(B)** HDAC4 Promoter Methylation Analysis: This section presents the frequency of HDAC4 promoter methylation as reported in other studies involving infarcted hearts. **(C)** HDAC6 Promoter Methylation Analysis: A heatmap representation shows the methylation levels of the HDAC6 promoter categorized by sample type and degree of methylation. **(D)** HDAC9 Promoter Methylation Analysis: This summary highlights the overall structure of the HDAC9 promoter, indicating the MAX binding hotspot and potential regulatory methylated regions. **(E)** MDM2 Promoter Methylation Analysis: This panel displays the methylation status of the MDM2 promoter, which varies depending on cell state or treatment conditions. **(F)** PIAS1 Promoter Methylation Analysis: Plots show the methylation status profile of the PIAS1 promoter and variations in methylation levels among different experimental groups. **(G)** PIAS2 Promoter Methylation Analysis: This section illustrates the methylation change points in the PIAS2 promoter. **(H)** RAN Promoter Methylation Analysis: This analysis presents the methylation status of the RAN promoter, including fractions of methylated sites and predicted regulatory effects. **(I)** RANBP2 Promoter Methylation Analysis: An overview of the methylation patterns in the RANBP2 promoter, identifying alterations that may negatively impact gene expression. **(J)** RANGAP1 Promoter Methylation Analysis: This panel highlights key intra-sample differences in DNA methylation for the RANGAP1 promoter. **(K)** SUMO1 Promoter Methylation Analysis: This section shows the distribution and extent of methylation in the SUMO1 promoter, which may play a role in gene regulation. The distribution of methylation across all sample types is visualized using bar graphs, with each wedge representing the proportion of sample types (Nb, CD34, and iPSC) containing CpG sites at low, medium, or high levels of methylation based on two different qMethylPlex assays. The rightmost column in each image displays the genome-wide distribution of ATAC peaks (rows), indicating chromatin accessibility relative to promoter methylation.

SUPPLEMENTARY FIGURE 2 Prognostic Model of SUMOylation-Related Genes for Glioblastoma (GBM) Prognosis. **(A)** To evaluate the diagnostic accuracy of ssGSEA score expressions in distinguishing tumor from normal groups, calibration curves were generated, accompanied by goodness-of-fit tests. The x-axis represents predicted probabilities, while the y-axis displays the actual rates. The red line indicates actual findings, and the blue dashed line reflects optimal predictions. According to the Hosmer-Lemeshow test, the P-value of 0.141 suggests inconclusive evidence, indicating that the model fits the data well. **(B)** A comparison of ssGSEA score expressions between tumor and normal groups is presented. A violin plot illustrates the distribution of ssGSEA scores (y-axis) for a selection of input gene sets, with the normal group shown in blue and the tumor group in red. No significant difference was observed between the two groups, as indicated by a p-value of 0.715. **(C)** The Receiver Operating Characteristic (ROC) curve evaluates the diagnostic accuracy of the ssGSEA score in differentiating between tumor and normal groups. The AUC is 0.540 with a 95% CI of 0.298–0.701, confirming the model’s diagnostic competence. **(D, E)** The colony formation assay indicates that Paromomycin affects the proliferation of glioma cells.
