# Supplementary figures and images for: Paromomycin targets HDAC1-mediated SUMOylation and IGF1R translocation in glioblastoma
Source: Front Pharmacol. 2024 Dec 11;15:1490878. doi: 10.3389/fphar.2024.1490878 (PMC11668589; doi:10.3389/fphar.2024.1490878)

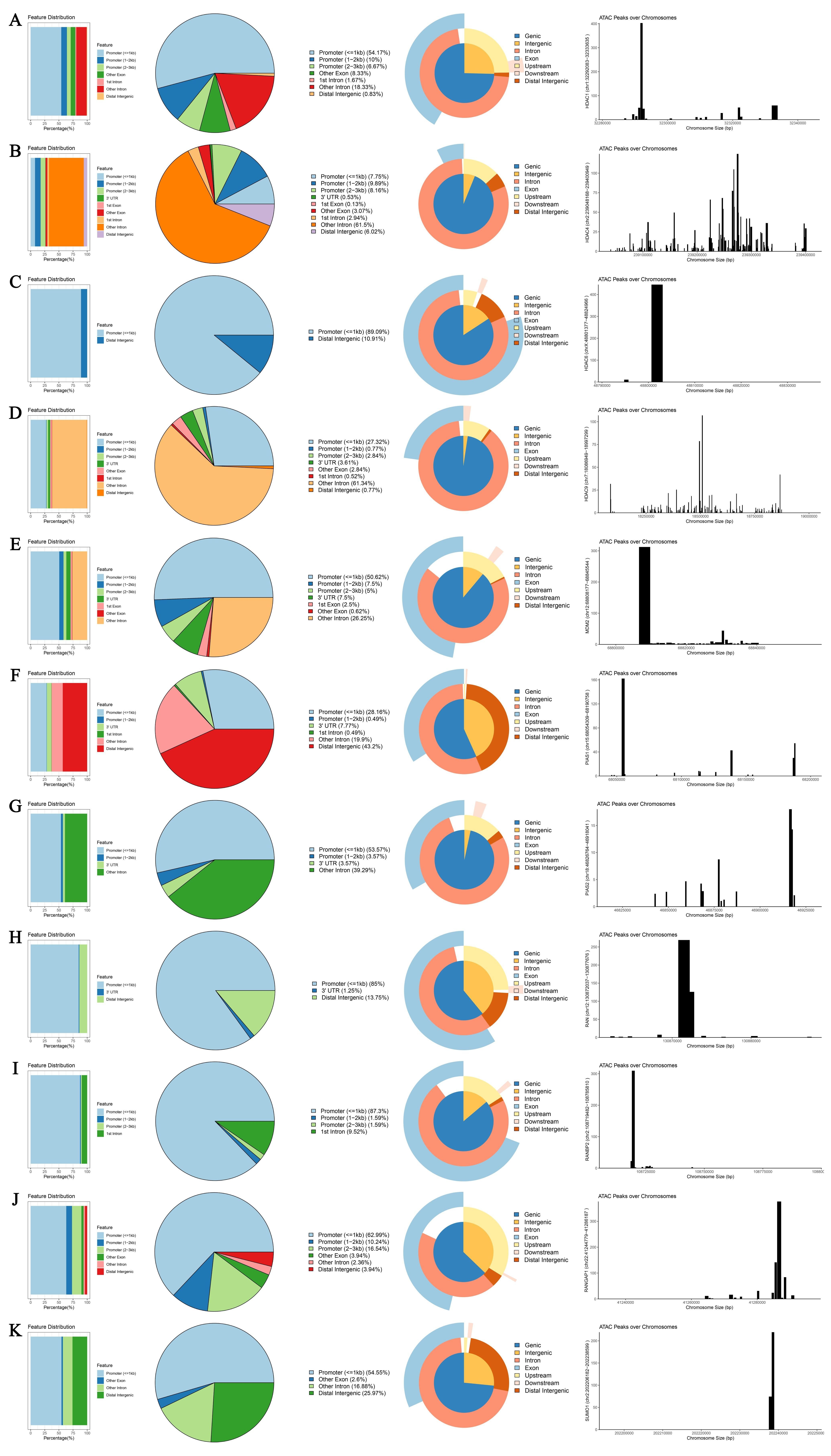

Supplement: Supplementary file 1 [file Image1.jpeg]

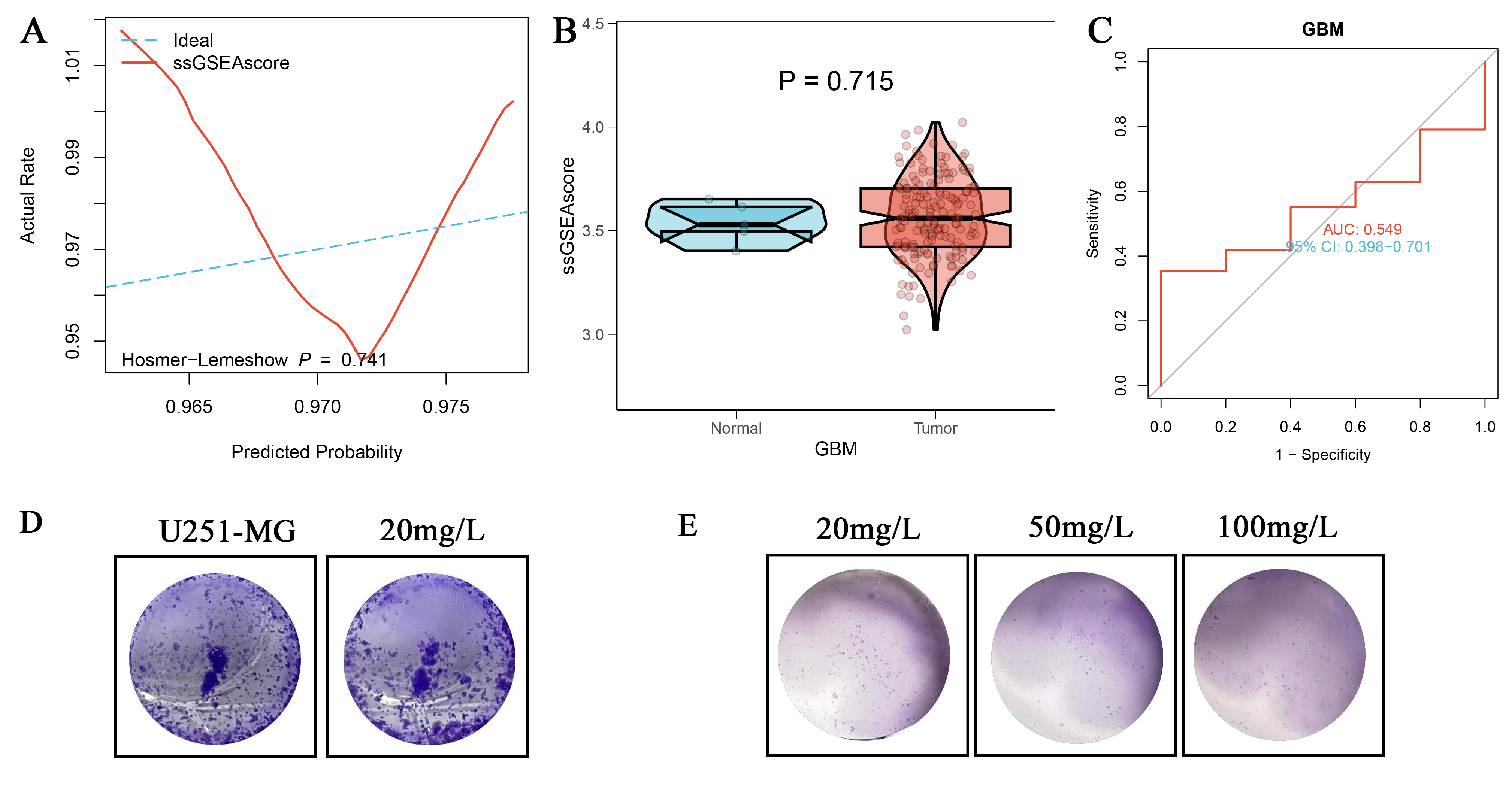

Supplement: Supplementary file 2 [file Image2.tif]
